# A computationally efficient algorithm for wearable sleep staging in clinical populations

**DOI:** 10.1038/s41598-023-36444-2

**Published:** 2023-06-06

**Authors:** Pedro Fonseca, Marco Ross, Andreas Cerny, Peter Anderer, Fokke van Meulen, Hennie Janssen, Angelique Pijpers, Sylvie Dujardin, Pauline van Hirtum, Merel van Gilst, Sebastiaan Overeem

**Affiliations:** 1 Philips Research Eindhoven, High Tech Campus 34, 5656AE Eindhoven, The Netherlands; 2 Department of Electrical Engineering, Eindhoven University of Technology, Eindhoven, The Netherlands; 3 Sleep and Respiratory Care, Philips Austria GmbH, Vienna, Austria; 4 Sleep Medicine Center Kempenhaeghe, Heeze, The Netherlands

**Keywords:** Medical research, Engineering

## Abstract

This study describes a computationally efficient algorithm for 4-class sleep staging based on cardiac activity and body movements. Using an accelerometer to calculate gross body movements and a reflective photoplethysmographic (PPG) sensor to determine interbeat intervals and a corresponding instantaneous heart rate signal, a neural network was trained to classify between wake, combined N1 and N2, N3 and REM sleep in epochs of 30 s. The classifier was validated on a hold-out set by comparing the output against manually scored sleep stages based on polysomnography (PSG). In addition, the execution time was compared with that of a previously developed heart rate variability (HRV) feature-based sleep staging algorithm. With a median epoch-per-epoch κ of 0.638 and accuracy of 77.8% the algorithm achieved an equivalent performance when compared to the previously developed HRV-based approach, but with a 50-times faster execution time. This shows how a neural network, without leveraging any a priori knowledge of the domain, can automatically “discover” a suitable mapping between cardiac activity and body movements, and sleep stages, even in patients with different sleep pathologies. In addition to the high performance, the reduced complexity of the algorithm makes practical implementation feasible, opening up new avenues in sleep diagnostics.

## Introduction

The detailed assessment of overnight sleep structure using polysomnography (PSG) remains a cornerstone of the diagnostic trajectory in sleep medicine. Based primarily on the characteristic patterns of electroencephalographic, electrooculographic and electromyographic activity, epochs of 30-s are assigned to wake, REM sleep or 3 classes of non-REM sleep of increasing ‘depth’ (N1–N3). A myriad of information is obtained from these measurements, for the assessment of overall sleep quality, the diagnosis of specific sleep disorders and the judgement of the severity or relevance of sleep-related events. The central position of PSG may even be considered on a higher level, as it forms an ‘obligatory passage point’ that structures and integrates subfields of sleep medicine through a common language^1^.

Despite its pivotal role, the use of PSG is hampered by a number of limitations. It is a labor-intensive procedure, from the data acquisition, annotation as well as interpretation perspective, suffering from well-known inter- and intraobserver variability. As such, it represents significant costs to healthcare and, given the increasing number of patients with sleep disorders, poses a higher demand on available diagnostic procedures. The PSG measurement itself may influence the quality of sleep due the required measurement electrodes and leads, which may be one of the reasons for so-called first-night effects. Finally, long-term monitoring is not possible using the standard PSG procedures.

In recent years, significant technological advances have yielded new sensors that permit cheap, long-term, and non-obtrusive recording of a range of biosignals. These allow for a new approach to sleep staging, based on the analysis of patterns in body movements and autonomic nervous system changes that occur between the various phases of sleep. This is the rationale behind the avalanche of consumer sleep trackers that to some degree allow the assessment of sleep–wake patterns, in healthy individuals. Further developments in machine learning and artificial neural networks, combined with newly available patient datasets containing gold standard PSG together with the new sensors, have indeed resulted in surrogate sleep staging approaches with substantial performance for (simplified) sleep staging^2^. For example, we recently reported on such an approach using wearable actigraphy and PPG-based heart rate variability to perform 4-class sleep stage classification, being able to distinguish between Wake, combined N1 and N2, N3 and REM sleep. With an epoch-per-epoch Cohen’s κ coefficient of agreement of 0.62, the technology is starting to approach human interscorer agreement, with small biases in the estimation of overall sleep statistics, even on a cohort of patients with sleep disorders^3^. However, an important drawback hampering the practical implementation of many of these algorithms, is their reliance on a large number of manually engineered features and the associated computational complexity. This requires off-line computation power and time, rendering these approaches unscalable to usage in larger cohorts. Neural networks, in particular applied to the supervised—but automatic—learning of feature representations may offer a solution, due to their relative computational efficiency during inference. As such, these are more and more deployed in real-world systems, even in embedded or smartphone implementations with dedicated chipsets. In the present study we describe such a new computationally efficient algorithm using an end-to-end deep learning approach, which allows 4-class sleep staging in patients with various sleep pathologies. We compare it with previous work in terms of performance in respect to agreement with manually scored sleep stages from PSG, as well as in terms of execution speed.

## Methods

### Datasets

This study made use of two separate datasets; the training set was used to train the sleep stage classifier algorithm and comprised PSG and accompanying PPG/accelerometer recordings that were not further used in validation. The hold-out validation set comprised PSG and accompanying PPG/accelerometer recordings that were not, in any point, used to train, tune, or in any way modify any algorithm. These were therefore used to have an unbiased view on performance for the end points of the study.

**Figure 1:**
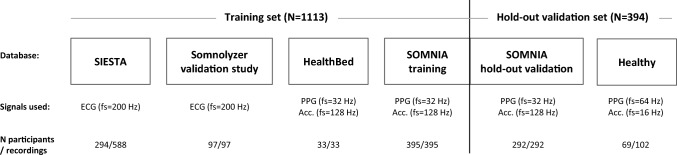
Distribution of recordings from each database into training and hold-out validation sets, with an indication of the number of participants and recordings, and signals used in the study with the corresponding sampling frequency (fs) (ECG, Electrocardiogram, PPG, Photoplethysmogram, Acc., Accelerometer).

Figure 1 illustrates how the databases were split between the training and the hold-out validation sets.

To characterize both sets, we calculated sample statistics describing demographics (sex, age, BMI) and sleep statistics based on sleep stage scorings obtained from PSG (total sleep time, sleep onset latency, wake after sleep onset, percentage of N1, N2, N3 and REM—all relative to total sleep time—and sleep efficiency). Differences in the values of each statistic between the training and the hold-out validation set were further compared for significance using a Mann–Whitney U test, as were differences between the recordings of SOMNIA (which were split between training and hold-out validation). A Bonferroni correction for 20 repeated tests was used, and the target *p*-value for significance was established at 0.05/20 = 0.0025.

### Training set—SIESTA

The training set comprised a combination of four different data sources, with a total of 1113 recordings. The first source was the SIESTA database^4^ which contains two PSG recordings for each participant, with 394 recorded from 197 healthy participants and 194 recorded from 97 patients. The participants were all adults with ages between 20 and 95 years. Patient diagnoses included sleep disorders such as sleep disordered breathing and periodic limb movement disorders; psychiatric disorders like anxiety and depressive disorders; and medical disorders such as Parkinson’s disease. Recordings were acquired from seven clinical partners and the study protocol was approved by each institutional review board individually. Consent was obtained prior to participation for each participant. Sleep stages were manually scored according to the Rechtschaffen & Kales (R&K) scoring rules^5^ by two independent experts and an additional consensus scorer out of a pool of 30 experienced sleep technicians.

### Training set—Somnolyzer validation study

The second source was a database of 97 PSGs originally recorded for the purpose of validating the Somnolyzer auto-scoring system^6^. The recordings were obtained from three sleep laboratories performing routine PSGs including diagnostic, positive airway pressure titration, and split nights. The study protocol was approved by the institutional review board of each clinical site and consent was obtained prior to the overnight session of each participant. Sleep stages were scored according to the AASM criteria published in 2007^7^ by four independent experts.

### Training set—SOMNIA and HealthBed

The third source used in the training dataset comprised a randomly selected set of sleep disordered patients, part of the Sleep and OSA Monitoring with Non-Invasive Applications (SOMNIA) study^8^ collected at the Sleep Medicine Center Kempenhaeghe between January 1, 2017 and July 24, 2018.

The fourth and last source used in the training dataset comprised recordings of healthy adult participants, without any sleep disorders or other medical or psychiatric comorbidities, acquired from the so-called ‘HealthBed’ dataset, also collected at the Sleep Medicine Center Kempenhaeghe^9^. This subset included data recorded before July 18, 2018. Both the SOMNIA and HealthBed studies were reviewed by the medical ethical committee of the Maxima Medical Center (Veldhoven, the Netherlands; file no: N16.074 and W17.128). All studies met the ethical principles of the Declaration of Helsinki, the guidelines of Good Clinical Practice and the current legal requirements. Consent was obtained prior to the overnight date for each participant, in case of adults, or the guardian, in case of children or adolescents. The protocols for the data analysis described in the present study were approved by the Medical Ethical Committee of the Kempenhaeghe hospital and by the Internal Committee of Biomedical Experiments of Philips Research.

For both the SOMNIA and the HealthBed studies each PSG was recorded during an overnight diagnostic study at Sleep Medicine Center Kempenhaeghe, using the recommended set of AASM channels in combination with a wrist-worn, CE-marked PPG and accelerometer sensor, built into a watch-like device with a silicon wrist strap^10^. The PPG and accelerometer sensors are an early prototype of the same sensors used in the Philips Healthband device (Royal Philips, Amsterdam, The Netherlands). The PPG sensor used two green light LED sources, and the signal was sampled at 32 Hz; the triaxial accelerometer data was sampled at 128 Hz. Each PSG in the SOMNIA and the HealthBed studies was scored by a single scorer out of a pool of seven experienced certified technicians from the Sleep Medicine Center Kempenhaeghe (Heeze, the Netherlands). Sleep stages were scored by experience sleep technicians in 30-s epochs using the 2015 AASM criteria^11^.

### Hold-out validation set—SOMNIA

The hold-out validation dataset comprised a combination of two different data sources, with a total of 394 recordings. The first source was a separate subset of SOMNIA, comprising a randomly selected set of recordings that were not part of the training set, recorded before December 10, 2019. The protocol for data collection, and reference scoring was the same as the SOMNIA subset used for training. In addition, along with the PSG, PPG and accelerometer data of each patient, the primary—or multiple, where applicable—diagnosis were entered by the clinician after the diagnostic trajectory of each patient and coded according to the International Classification of Sleep Disorders, 2nd edition (ICSD-2)^12^. For sake of simplicity in the analysis, disorders were grouped per major category.

### Hold-out validation set—healthy

The second source set (referred to as ‘Healthy’ from here on) was collected between 2014 and 2015 and consisted of recordings of healthy, middle-aged participants on two consecutive nights in a hotel, which included overnight PSG and an earlier version of the CE-marked PPG and accelerometer prototype, where the PPG signal was sampled at 64 Hz and the triaxial accelerometer data at 16 Hz^13^. This study included participants with no primary history of neurological, cardiovascular, psychiatric, pulmonary, endocrinological, or sleep disorders. In addition, none of the participants were using sleep, antidepressant or cardiovascular medication, recreational drugs, excessive amounts of alcohol, nor were they pregnant or working in shifts, nor crossing more than two time zones in the 2 months prior to the investigation. All participants had a body mass index lower than 40 and a Pittsburgh Sleep Quality Index (PSQI) lower than 6. The study was approved by the Internal Committee of Biomedical Experiments of Philips Research and was conducted in accordance with the Declaration of Helsinki. All participants gave informed consent before participation. The hotel gave approval to conduct the experiment on their premises. In the case of this subset of the hold-out validation set, the PSG data were scored by an external certified experienced somnologist (SleepVision, Nijmegen, the Netherlands), and the sleep stages were manually scored in 30-s epochs according to the AASM guidelines^11^.

### Ground-truth PSG sleep stages

Since the sleep staging algorithm described in this study outputs a simplified hypnogram with four classes, the ground-truth reference classes were obtained from all data sources for both the training and the hold-out validation sets by simplifying the class labels. In the case of R&K scoring of the SIESTA database, S1 and S2 were combined in a single “N1–N2” class and S3 and S4 in a single N3 class, while the remaining classes (Wake, and REM) were used without changes. In the case of AASM scorings from all other databases, N1 and N2 were combined in a single “N1–N2” class while the remaining classes (Wake, N3, and REM) remained unchanged.

### Sleep stage classification

**Figure 2:**
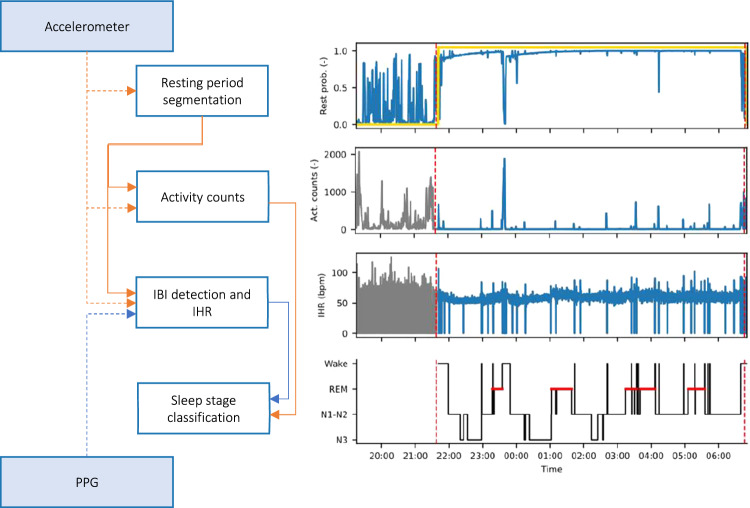
Overview of the processing steps for sleep stage classification based on accelerometer and PPG inputs; from top to bottom the plots represent: probability of the participant being at rest (in blue), and resting period found in the complete recording (in yellow), activity counts (in grey for the excluded period outside the resting period, in blue, during the resting period), instantaneous heart rate (idem), and predicted sleep stages; the vertical dashed red lines indicate the boundaries of the lights off period.

Figure 2 illustrates, with a block diagram, the different processing steps for the overall sleep stage classification algorithm. Using accelerometer information, periods where the participant is likely resting were first determined. After the activity counts and the interbeat intervals and corresponding instantaneous heart rate (IHR) signals were computed, samples outside the resting period were excluded from further processing. The resulting signals were used as input to first train the sleep staging model, using the training set, and then to validate it, using the hold-out set. The sleep staging model was configured to output a prediction of sleep stages for each 30 s epoch during the resting period.

It should be noted that not all subsets in the training set contain accelerometer and PPG data; in those cases, the complete recording was used, and interbeat intervals were calculated from the ECG signal included with the PSG. It should be emphasized that this was only the case with data in the training set and the strategy of combining the two cardiac sensing modalities served the sole purpose of having sufficient and varied data to train the model, given the lower availability of PSG recordings with accompanying wrist-worn PPG. All recordings in the hold-out validation set contained valid PPG and accelerometer data and were used as such. In other words: hold-out validation performance reflects how the model performed using PPG and accelerometer only.

### Resting period segmentation

In the SOMNIA and HealthBed studies, participants were fitted with the wrist-worn PPG/accelerometer sensors several hours before their bedtime. To limit the computation of sleep stages to the periods where the participants were resting, an algorithm was used to automatically segment resting periods, i.e., intervals during which the participant is not physically active, moving or walking. The algorithm, described in earlier work^14^, is based on the analysis of the tri-axial accelerometer data and it starts by computing 1 s-based motion characteristics such as number of zero crossings, periodicity, vertical acceleration, and motion cadence. These features are then aggregated into samples of 30 s from which the mean, standard deviation, maximum value and 95th percentile are calculated. A pre-trained Bayesian linear discriminant is then used to estimate, for each 30 s epoch, the likelihood that the participant was resting. All epochs for which the likelihood was below the pre-determined threshold of 30% were not analyzed by the sleep staging algorithm (by setting input data to zero); periods outside the main resting period (defined between the start of the first detected resting period, and the end of the last resting period) were simply marked as Wake in the final sleep staging output.

### Interbeat interval detection

One of the inputs to the sleep staging classification algorithm is a time series of IHR during the resting period (the IHR values outside the resting period are simply set to 0). Besides offering a detailed representation of cardiac activity, using IHR has the advantage of providing a layer of abstraction from the type of cardiac sensor (PPG, ECG) and sampling frequency used for data acquisition, provided that the signal can be used to reliably and accurate localize individual heart beats. This technique effectively enabled us to enrich our training set beyond only datasets with simultaneous recording of PPG and PSG (which are not abundantly available) and include PSG acquisitions where ECG is available.

IHR was calculated from the PPG signal by first detecting the location of individual beats (pulses) using an algorithm described in detail in earlier work^15^. In short, pulses are detected by identifying fiducial points (troughs) in the waveform, determined as the points where the first derivative crosses zero towards a positive value. Given the susceptible nature of PPG to the presence of motion artifacts, and to minimize the impact of inaccurate pulse detections, the maximum magnitude of acceleration measured with the accompanying accelerometer, was assessed every second. Local minima occurring during periods where a threshold of 0.1 g was exceeded were discarded. The time interval between consecutive PPG fiducial points was then used to represent the interbeat interval (IBI) time series. This time series was finally resampled to a fixed sampling frequency of 10 Hz, inverted and multiplied by 60 to obtain a representation of IHR (in beats per minute, bpm).

In the SIESTA and the Somnolyzer validation studies, all part of the training set, wrist-worn PPG data is not available. In the case of these recordings (which are used only for training) IHR was obtained after detecting heart beats with a QRS detector^16^, followed by a post-processing localization algorithm^17^. IHR was calculated in a similar manner as for PPG.

### Activity count estimation

The second input to the sleep stage classification algorithm is a time series of activity counts representing gross body movements, obtained with an algorithm described in detail in earlier work^18^. In short, activity counts are computed for each 30 s based on the three-axial accelerometer signal of the wrist-worn device. Each accelerometer axis is first low-pass filtered with a third-order Butterworth filter and a cutoff frequency of 1 Hz. A simple estimate of gravity is then computed for each nonoverlapping 1 s window as the average amplitude of each axis in that window. A 1 s activity count value is computed as the sum of the absolute values of each axis, minus the estimate of gravity for that window. To obtain a representation of activity counts for each 30 s epoch, all 1 s values inside each 30 s window are simply added. Unlike for the IHR, activity counts were not set to 0 outside the resting periods. Since the participant is moving, the classifier can use this information to interpret these periods as probable Wake.

### Clock synchronization

To allow an epoch-per-epoch comparison between the predicted and the reference ground-truth sleep stages, the clock of the PPG/accelerometer sensor was synchronized, for each recording, with the clock of the PSG, using the ECG signal recorded as part of all PSG acquisitions. Using a QRS detector^16^, followed by a post-processing localization algorithm^17^, an IBI time series was first computed for the ECG signal in the PSG recording. The clock offset and drift were determined by maximizing the correlation between the ECG-derived IBI series and the PPG-derived IBI series described before.

### Classifier

In previous work, we built a model based on 132 manually engineered ECG-based HRV features (ECG–HRV)^18,19^ which we then applied directly on HRV features extracted from PPG recordings of a hold-out set^20^. This led to a decrease in performance, which we resolved by training a similar model but now, based on HRV features extracted directly from PPG (PPG–HRV)^3^. In the present study we used this model as is, without retraining, to serve as baseline for performance and run time comparison. Using as input the IBI time series derived from PPG, and the activity counts from the accelerometer, we calculated sleep stages for all recordings of the hold-out validation set.

As the main objective of this study, we also developed a new end-to-end neural network sleep stage classifier (PPG–NN) which instead of manually engineered features, uses as input IHR and activity counts, and where feature extraction is achieved with a set of convolutional neural network layers which can learn adequate representations of the data for this classification task. IHR was interpolated at 10 Hz, and the activity counts were used as computed, with a sampling rate of one sample per 30 s, i.e., approximately 0.033 Hz.

**Figure 3:**
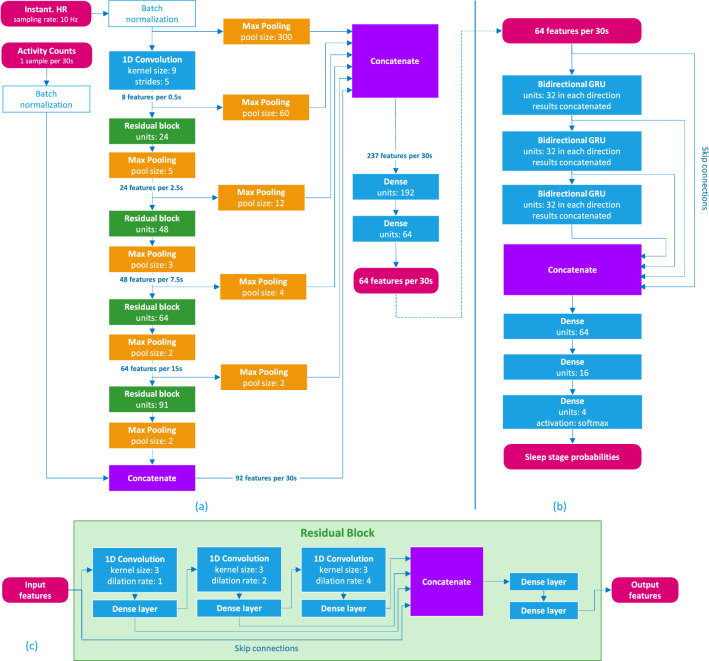
Architecture of the sleep staging neural network. Panel (**a**) illustrates the overall architecture of the feature extraction network. Residual convolutional blocks with increasing number of outputs are combined with maximum pooling layers in an alternating fashion to gradually increase the complexity of extracted features and at the same time reduce the temporal resolution from the 10 Hz input to one feature vector per 30 s. Skip connections are added after each step. Panel (**b**) shows the architecture of the recurrent sleep stage classifier. Bidirectional GRU layers are stacked and skip connections ease the learning of simple relationships by bypassing the recurrent layers. Panel (**c**) shows the architecture of the residual convolutional blocks used in the feature extraction network. Convolutional layers are combined with additional dense layers to increase the network depth without increasing the perceptive field of the convolutional block.

The architecture of the neural network comprises two parts. The first, a convolutional feature extraction network (Fig. 3, panels (a) and (c)), was built from a series of residual convolutional blocks and pooling layers that gradually decrease the temporal resolution while increasing the richness and complexity of the extracted features until it finally outputs a 64-dimensional feature-vector per 30 s. The residual blocks were inspired by encoder networks that stack one-dimensional convolutional layers with increasing dilation rates and add skip connections^21,22^. When stacking many convolutional layers with increasing dilation rates, the perceptive field (the number of time steps in the input data that determines a single time step in the output) can grow very large easily, possibly leading to a model overfit to the training data. To achieve a high level of complexity and still limit the perceptive field of the feature extraction network to locally relevant data, the convolutional layers were combined with dense layers which could be interpreted as convolutions with a kernel length of one. This technique is frequently used in image classification and segmentation tasks by using convolutional layers with a single pixel (1 × 1) convolutional kernel^21–25^. Skip connections were used throughout the network to avoid the vanishing gradients problem during training and to enable the learning of simple and complex features by bypassing some of the layers^26^. We used the scaled exponential linear unit (SELU) activation function^27^ throughout the convolutional part of the neural network to take advantage of the resulting network’s self-normalizing properties during training. To preserve the self-normalizing properties of this activation function, skip connections are concatenated rather than adding them to the outputs of bypassed layers.

The 64 features output by the feature extraction block are then fed into the second part (Fig. 3, panel b), a recurrent neural network which uses bidirectional layers of gated recurrent units (GRUs)^28^ for feature harmonization and sleep stage classification. Three bidirectional GRUs are stacked and skip connections bypassing the recurrent layers are concatenated to the output. The recurrent layers can use global context from the entire acquisition to normalize features and to model human sleeping patterns. Finally, a fully connected layer with 4 outputs and a softmax activation function predicts probabilities for the sleep stages Wake, N1–N2, N3, REM.

The hyperparameters of the network, and in particular, the kernel sizes of the convolutions were chosen such that the perceptive field of the feature extraction would be comparable to the size of the windows used to extract HRV features in the baseline PPG–HRV algorithm. The number of extracted features, size and number of the recurrent units was chosen to be similar to a previously published successful sleep classification network using cardio-respiratory input signals^29^. The model was trained using the categorical cross entropy loss function without sample, or class weighing, since in our experience, this loss function can adequately handle small class imbalances. In our dataset, these don’t exceed a factor of 1 (for the class with lowest prevalence, N3) to 4 (for the class with the highest prevalence, combined N1–N2) and importantly, there are sufficient positive examples of each class in the training set such the gradient updates during back-propagation are not dominated by the “easier” examples of the dominant class.

### Performance evaluation

After synchronizing the clocks of the PPG/accelerometer and the PSG as described before, and after performing sleep staging based on the PPG/accelerometer recordings, we then restricted the analysis of performance to the “lights off” period for which the PSG sleep stages were scored. After sleep stage classification, epoch-per-epoch agreement between the predicted classes and the ground-truth PSG sleep stages was evaluated using metrics of accuracy and Cohen’s κ coefficient of agreement (or κ). Accuracy measures the percentage of 30 s epochs with correctly classified sleep stages for each recording and κ measures performance after correcting for agreement by chance. Given the imbalance between classes (N1–N2 is more prevalent than either of the other classes, often corresponding to more than 50% of the epochs in a sleep recording), κ is more informative, and usually interpreted as follows: below 0.20 “slight agreement,” between 0.20 and 0.40 “fair agreement,” between 0.40 and 0.60 “moderate agreement,” between 0.60 and 0.80 “substantial agreement,” and above 0.80 “almost perfect agreement”^30^.

Both metrics were computed for four classes, three classes (merging N1–N2 and N3 in a single non-REM “NREM” class), and two classes (merging N1–N2, N3 and REM in a single “Sleep” class). In the case of two classes, we calculated in addition also the sensitivity, specificity and positive predictive value (PPV), all considering “Wake” as positive class. A similar analysis was done for the remaining classes, each time considering each class as positive in comparison with the remaining, merged in a single negative class.

To evaluate the possible relation between different demographic variables like sex, age or BMI and performance, we fitted a beta regression^31^, particularly adequate to model proportions, in this case κ, bounded between 0 and 1; the link function was empirically chosen as the one that maximized the pseudo-R^2^ ^31^ of the resulting fit. We also included terms that could plausibly explain differences in performance such as the percentage of epochs in each recording for which valid IHR was available (“Coverage”), percentage of epochs with activity counts above 40 (“Act > 40”; the threshold was chosen as the same used in previous work to classify Wake and Sleep based on actigraphy^13^), and apnea–hypopnea index available as a clinical variable for the participants in the SOMNIA subset (AHI). This evaluation was performed for the complete hold-out validation set, and for any disorder sub-groups with a significantly lower performance.

Throughout the manuscript a Shapiro–Wilk normality test was used to determine whether the sample data and validation statistics were normally distributed (at a *p*-value of 0.05). In that case, the descriptive statistic indicates the mean, and between parenthesis, the standard deviation (SD): “mean (SD)”. When the data was not normally distributed, the statistic indicates the median, and between curly brackets, the 25th and 75th percentiles: “median {Q1, Q3}”.

### Run time and performance comparison

The ECG–HRV and PPG–HRV classifiers described in previous work both relied on many manually engineered features, some of which with rolling windows of a large size (such as multiscale sample entropy calculations over 8.5 min of IBI data), others with non-linear complex computations (such as detrended fluctuation analysis, or visibility graphs).

The algorithm developed in the present study (PPG–NN) did not rely on manually engineered features, but instead on a neural network that learned adequate feature representations, suitable for this classification task. To test whether this approach represented an improvement in run time performance compared to the PPG–HRV model, we timed the complete processing time per recording, for all recordings of the hold-out validation set. This timing included the loading of the raw PPG and accelerometer data, the detection of IBIs and activity counts, and in the case of the PPG–NN model, calculation of IHR and inference, and in the case of the PPG–HRV model, extraction of all 132 features, and inference. To gain further insights into the run time of the separate parts of both algorithms, we chose the recording with a run time closest to the median for the PPG–HRV classification. We then profiled the run time of the different HRV feature extraction blocks for the PPG–HRV model, and evaluated the complexity of the neural networks used by each algorithm by estimating the number of multiply-accumulation (MAC) operations using STM32CubeIDE version 1.12.0 (STMicroelectronics, Plan-les-Ouates, Switzerland). All analyses ran on the same machine, with an Intel Xeon W-1250P CPU at 4.10 GHz.

In principle, the PPG–NN algorithm should represent a run time performance improvement over the PPG–HRV algorithm. However, that should not come at the cost of sleep stage classification performance. To verify whether that was the case, we used an equivalence test for sleep stage classification agreement (using epoch-per-epoch κ) between the predictions obtained with each algorithm, and the reference from PSG, using a confidence interval (CI) of 95%^32^. Since the performance of neither algorithm followed a normal distribution, the CI was determined with a non-parametric method based on Wilcoxon’s signed rank test^33^. Equivalence was established if the 95% CI for difference in performance using PPG–NN and PPG–HRV was fully contained in the interval established by a predefined margin [− δ, δ]. Since there is, to the best of our knowledge, no established margin described in literature for similar comparisons, we followed the recommendations of Walker and Nowacki^32^ and chose as δ the lower bound of the 95% CI for difference in performance between both of our previous HRV models, namely between PPG–HRV and ECG–HRV. In other words: if the 95% CI of the difference in performance between PPG–NN and PPG–HRV is fully contained in the margin defined by the difference in performance between PPG–HRV and ECG–HRV, we consider PPG–NN to yield an equivalent performance to the HRV algorithms.

### Ethical parameters

The study was approved by EC/IRB (Medical Ethical Committee of the Maxima Medical Center, Veldhoven, the Netherlands; Internal Committee of Biomedical Experiments of Philips Research, Eindhoven, the Netherlands; Medical Ethical Committee of the Kempenhaeghe hospital, Heeze, the Netherlands) and study was conducted in accordance with the Declaration of Helsinki. All adult participants, or in the case of children and adolescents, their guardian, gave informed consent before participation.

## Results

### Demographics and clinical information

Table 1 and supplementary Table S1 indicate the demographic information and PSG-derived sleep statistics for the participants in the training and in the hold-out validation sets. Age, total sleep time, wake after sleep onset, percentages of N2 and N3 and sleep efficiency were significantly different between the training and the hold-out validation recordings. However, when comparing only the SOMNIA subsets, since these were randomly sampled between training and hold-out validation, only age (owing to the inclusion of more children and adolescents in the hold-out validation group) and total sleep time were significantly different.

**Table 1:** Demographic information of participants in the training and hold-out validation datasets.

Dataset	Participants/recordings	N Female (%)	Age (year)	BMI (kg/m^2^)
**Training**	819/1113	449 (40.3)	50.9 (16.8) [3, 95]	25.8 {23.0, 28.8} [16.5, 46.1]
SIESTA	294/588	252 (42.9)	51.5 (17.3) [20, 95]	25.1 {22.6, 27.7} [16.5, 43.3]
Somnolyzer validation study	97/97	33 (34.0)	55.9 (14.7) [22, 80]	n/a
SOMNIA training	395/395	146 (37.0)	53 {40, 61} [3, 86]	27.2 {24.3, 30.0} [16.8, 46.1]
*Adults*	388/388	144 (37.1)	53 {41, 61} [18, 86]	27.2 {24.3, 30.0} [16.8, 46.1]
*Children/adolescents*	7/7	2 (28.6)	10.7 (5.71) [3, 17]	n/a
HealthBed	33/33	18 (54.5)	24 {20, 50} [18, 63]	24.1 (2.84) [18.4, 30.4]
**Hold-out validation**	352/394	168 (42.6)	47 {33, 58} [3, 82]*	26.2 {23.8, 29.3} [17.5, 48.9]
SOMNIA hold-out	292/292	106 (36.3)	45 {27, 58} [3, 82]^+^	26.2 {23.9, 29.7} [18.5, 48.9]
*Adults*	244/244	86 (35.2)	49 {36, 60} [19, 82]	26.2 {23.9, 29.7} [18.5, 48.9]
*Children/adolescents*	48/48	20 (41.7)	13 {9, 15} [3, 17]	n/a
Healthy	60/102	62 (60.8)	51 {45, 58} [26, 66]	25.8 (3.91) [17.5, 36.2]

Descriptive statistics indicate mean and between parenthesis, SD in case of normally distributed data, or median and between curly brackets, 25th and 75th percentiles otherwise; the range is indicated between square brackets in both cases.

BMI is not available for the Somnolyzer validation study nor for the children/adolescents in SOMNIA and was omitted from calculation of sample statistics. Rows with ‘Training’ and ‘hold-out validation’ datasets (in bold) indicate the aggregated statistics from all recordings on the corresponding subsets. Separate statistics are also provided for each subset and in case of the SOMNIA datasets, further statistics are differentiated between adults and children/adolescents (in italic).

BMI, Body mass index. *, ^+^: Significant difference between hold-out validation and training set, and ^+^between SOMNIA subset in the hold-out and SOMNIA subset in the training sets, after a Mann–Whitney U test with Bonferroni correction for 20 repeated tests (target *p*-value for significance = 0.0025).

Table 2 indicates the prevalence of each major category in the hold-out validation cohort, and an indication of the number of participants for whom each category was the single primary diagnosed disorder.

**Table 2:** Prevalence of each disorder category in the hold-out validation set, and number of participants for whom each disorder was the single primary diagnosis in the hold-out validation cohort.

Group	Prevalence	Single primary disorder
Sleep disordered breathing	120	99
Insomnia	75	49
Movement disorder	29	15
Behavioral	20	11
Non-REM parasomnia	15	13
REM parasomnia	10	5
Other	29	17
None	101	n/a

A total of 8 children/adolescents (out of 48) had no sleep disorder.

### Sleep staging performance

**Figure 4:**
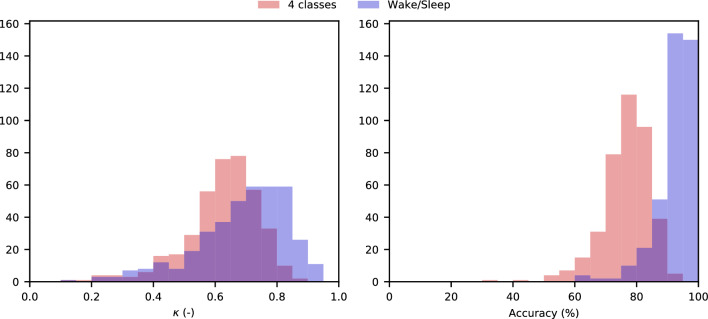
Histograms with (left) κ and (right) accuracy for (red) 4-class and (blue) Wake/Sleep stage classification in the hold-out validation set; overlapping portions of the histograms are represented in purple.

Figure 4 illustrates histograms with the classification performance for 4-class and Wake/Sleep classification for the complete hold-out validation set of 394 recordings. A total of 94.9% of all recordings achieved a κ higher than 0.4 for 4-class classification, the lower threshold for moderate agreement.

Table 3 indicates the performance for different classification tasks, with the most challenging task, 4-class classification, achieving a median κ with substantial agreement of 0.638, and a median accuracy of 77.8%. When the task is simplified, the performance obviously improves to a median κ of 0.716 for 3 classes, and 0.712 for Wake/Sleep classification. In addition, the table indicates the performance for different binary tasks, where each of the three remaining classes (N1–N2, N3 and REM) were evaluated in a one-vs-rest scenario, with each class being considered as positive, and the remaining aggregated classes as negative. The best performance is achieved for REM classification, with a median κ of 0.751, while the lowest performance was obtained for N1–N2, with 0.561.

**Table 3:** Sleep stage classification performance for several classification tasks in the hold-out validation set (N = 394 recordings).

Task	κ (−)	Accuracy (%)	Sensitivity (%)	Specificity (%)	PPV (%)	F1 (%)
Wake/N1-2/N3/REM	0.638 {0.572, 0.704}	77.8 {73.2, 81.8}	n/a	n/a	n/a	n/a
Wake/NREM/REM	0.716 {0.633, 0.781}	87.4 {83.6, 90.3}	n/a	n/a	n/a	n/a
Wake/Sleep	0.712 {0.608, 0.799}	93.8 {90.7, 96.1}	78.7 {67.0, 87.8}	97.0 {94.3, 98.5}	79.7 {67.6, 88.9}	75.7 {66.7, 84.1}
N1–2	0.561 {0.468, 0.631}	78.7 {73.9, 82.2}	85.3 {78.8, 89.3}	72.9 {65.9, 79.6}	77.1 {68.1, 83.9}	79.9 {73.9, 83.9}
N3	0.637 {0.478, 0.744}	91.0 {87.3, 93.7}	66.8 {50.6, 85.7}	97.4 {94.4, 99.1}	82.0 {65.3, 94.8}	69.6 {56.0, 78.9}
REM	0.751 {0.629, 0.819}	94.0 {91.7, 95.9}	77.5 {62.1, 85.7}	97.6 {96.1, 98.9}	83.5 {74.4, 91.6}	79.1 {69.3, 85.0}

Almost none of the variables were normally distributed, so all descriptive statistics in the table indicate the median and between curly brackets, 25th and 75th percentiles.

PPV, Positive predictive value (equivalent to precision); F1, F1 score, harmonic mean of PPV (or precision) and sensitivity (or recall).

Table 4 indicates the confusion matrix for the classification of all pooled epochs in the hold-out validation set. Most confusion occurs between N1 and N2 and the remaining classes, especially with N3: 6.1% of all epochs were misclassified as N1–N2 while the reference indicated N3. The more implausible combinations (confusion between any combination of N3, Wake and REM) represent no more than 0.3% of all classifications.

**Table 4:** Confusion matrix for the classification of all pooled epochs in the hold-out validation set (N = 393,012 epochs).

pred. → ↓ref	Wake	N1–N2	N3	REM	Prev. (−, (%))	Sens. (%)	κ (−)
Wake	53,076 (13.5%/76.4%)	15,179 (3.9%/21.8%)	216 (0.1%/0.3%)	1018 (0.3%/1.5%)	69,489 (17.7%)	76.4	0.73
N1–N2	12,657 (3.2%/6.4%)	165,414 (42.1%/83.1%)	12,901 (3.3%/6.5%)	8015 (2.0%/4.0%)	198,987 (50.6%)	83.1	0.55
N3	549 (0.1%/0.8%)	23,895 (6.1%/36.2%)	41,326 (10.5%/62.7%)	158 (0.0%/0.2%)	65,928 (16.8%)	62.7	0.63
REM	1224 (0.3%/2.1%)	15,080 (3.8%/25.6%)	509 (0.1%/0.9%)	42,007 (10.7%/71.4%)	58,820 (15.0%)	71.4	0.72
PPV (%)	78.6	75.3	75.2	82.0			

Each entry in the confusion matrix indicates the number of epochs, and between parentheses, the percentage relative to the total number of epochs of all classes followed by the percentage relative to the total number of epochs with the corresponding reference sleep stage for that row. Prev., prevalence; Sens., sensitivity; PPV, positive predictive value.

Note that PPV, sensitivity and κ per class differ versus what was presented in Table 3: whereas the previous results described the sample statistics per participant, in the present table they represent the performance after aggregating all epochs from all participants.

Table 5 indicates the performance on the hold-out validation set for each disorder category, and the result of a Wilcoxon signed-rank test to compare each group against the remaining participants (without that disorder). With a Bonferroni correction for 7 tests, we established as target *p*-value 0.05/7 = 0.0071. The highest performance was achieved for the non-REM parasomnia group (not significantly higher than for all participants without that condition), with an average κ of 0.690, followed by the healthy participants (‘None’) with 0.657 (also not significantly higher than for participants with sleep disorders). The worst-performing group was for patients with movement disorders, with a median κ of 0.591 (not significantly worse than for patients without that condition). Supplementary Figure S1 illustrates examples comparing reference hypnograms from PSG with predicted hypnograms with the PPG–NN algorithm, for the participant closest to the median performance of each sleep disordered category.

**Table 5:** Performance on 4-class sleep stage classification on the hold-out validation set, for each disorder category.

Condition	N	κ (−)	*p*-value	Accuracy (%)	*p*-value
Sleep disordered breathing	120	0.621 {0.549, 0.687}	0.053	0.779 {0.731, 0.814}	0.99
Insomnia	75	0.625 (0.122)	0.92	0.772 (0.0702)	0.8
Movement disorder	29	0.591 {0.519, 0.643}	0.0083*	0.742 {0.717, 0.805}	0.035*
Behavioral	20	0.617 (0.108)	0.84	0.774 (0.0674)	0.63
Non-REM parasomnia	15	0.690 (0.0902)	0.028*	0.806 (0.0544)	0.043*
REM parasomnia	10	0.595 {0.589, 0.617}	0.22	0.757 (0.0217)	0.22
None	143	0.657 {0.585, 0.705}	0.11	0.780 {0.734, 0.809}	0.6

Note that because some patients had multiple primary sleep disorders diagnosed, the groups (except for ‘None’) are not mutually exclusive and the total N adds up to more than the size of the hold-out set (N = 394 recordings).

*Differences (κ, accuracy) between participants with a given disorder and participants without that disorder, with a *p*-value below 0.05; no differences were significant after a Bonferroni correction for 7 tests.

In this table, ‘None’ indicates the number of recordings, and not participants; note that for the subset with healthy participants there could be more than one recording per subject.

Descriptive statistics indicate mean and between parenthesis, SD in case of normally distributed data, or median and between curly brackets, 25th and 75th percentiles otherwise.

Supplementary Table S4 in indicates the coefficients and corresponding *p*-values for each term included in the beta regression fit to the κ obtained for the entire cohort, and for the sub-group with the lowest performance, i.e., with movement disorders. Given the repeated nature of the statistical tests, we used a Bonferroni correction for 6 terms to establish the target *p*-value of 0.05/6 = 0.0083. Accordingly, the only significant term for the entire cohort was age, suggesting that sex and BMI do not significantly explain performance. Coverage and percentage of epochs with activity counts above 40, were also not significant, and neither was AHI as an explanatory variable. Males had a slightly lower (but according to the model, not significantly so) median κ of 0.621 when compared to females, with 0.653. Supplementary Table S5 indicates the performance per age group, with the highest performance achieved for adolescents with a median κ of 0.684, significantly higher than for the remaining participants; the group with children achieved a mean κ of 0.650 (but not significantly different) and adults achieved a median κ of 0.632. In the sub-group analysis for movement disorders, both coverage and presence of body movements explain the performance, but age does not. The inclusion of these two terms (in addition to age) yields a pseudo R^2^ of 0.565 for this sub-group.

### Run time and performance comparison

Supplementary Figure S2 illustrates histograms with the run time of the PPG–HRV and the PPG–NN sleep stage classifiers for each participant in the hold-out set. The PPG–HRV algorithm ran for a median (25th, and 75th percentile) time of 10.9 (9.61, 12.5) minutes per recording, while the PPG–NN algorithm ran for a median of 0.206 (0.127, 0.223) minutes per recording. Supplementary Table S2 indicates the run time for different feature extraction and inference tasks for the PPG–HRV algorithm, excluding the pre-processing steps of data loading and pre-processing, activity count and IBI estimation since these are similar between the two methods. The HRV feature extraction steps take up 99.5% of this run time, with a main contribution from the extraction of frequency domain, sample entropy and detrended fluctuation analysis features.

Regarding the number of MAC operations, the PPG–NN neural network uses a total of 89.5 million operations per hour of recording, with 79.1 million operations spent in the ‘feature extraction’ (corresponding to the blocks in Fig. 3a), and 10.5 million operations in the recurrent parts of the neural network (GRUs, in Fig. 3b). In comparison, the PPG–HRV neural network, which consists mainly of recurrent neural units (LSTMs)^19^, uses a total of 30.9 million operations per hour of recording.

**Figure 5:**
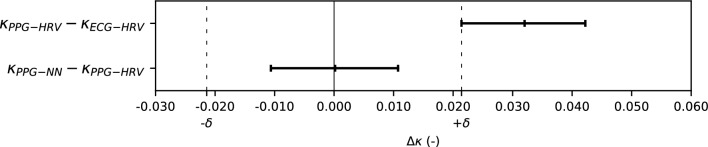
95% confidence intervals for difference in performance (above) between the PPG–HRV and ECG–HRV models, with the lower bound to establish δ and (below) between the PPG–NN and PPG–HRV model. Statistical equivalence in performance was established between the PPG and NN and the PPG–HRV models.

Figure 5 illustrates the 95% CI for difference in performance between the PPG–HRV and the ECG–HRV model; the lower bound of this CI (0.021) was used as margin δ for equivalency testing between PPG–HRV and PPG–NN, for which the 95% CI, [− 0.011, 0.011], is also illustrated in the figure; since the CI is completely contained within the margin defined by [− δ, δ], the performance of the two algorithms can be considered statistically equivalent.

## Discussion

The domain of automatic sleep staging based on autonomic nervous system activity has seen substantial advances in recent years, mainly driven by a better understanding of how HRV changes throughout sleep^34,35^. In previous work we used more than one hundred hand-crafted HRV features and showed how these could be used to accurately differentiate between wake, combined N1 and N2, N3 and REM in a cohort of patients with multiple (and often comorbid) sleep disorders^3^. In the present study, we approach the problem from a different angle: instead of hand-crafted features, we let a neural network learn suitable feature representations using as input only instantaneous heart rate, and activity counts. This approach yielded a much faster computation speed, while not reducing sleep staging performance compared to our previous approach, which has important implications.

First, this independently validates the principle of HRV—and actigraphy-based sleep staging and helps more firmly establish these as valid sensor modalities for this task, also in clinical populations with a wide age range and with a variety of sleep disorders. Although the starting point is the same for both algorithms, i.e., a gross measure of body movements measured with an accelerometer, and interbeat intervals—in our study—calculated from wrist-worn PPG, the neural network does not leverage any a priori knowledge of the domain, letting the task be instead completely driven by the automated “discovery” of the most suitable mapping between the input cardiac activity and the output sleep stages.

Second, this offers a much more attractive approach from a practical product implementation perspective, in actual wearable devices, or in associated cloud computing services.

Both algorithms have feature extraction modules which operate on a moving-time windows of fixed size, albeit one uses manually engineered features, and the other, a stack of convolutional neural network units. The computational complexity of both algorithms is thus linearly dependent on the number of epochs and the duration of the recording. Furthermore, due to the bidirectional recurrent computation of sleep stages in both algorithms, the inference time delay is equivalent to the duration of an entire recording in both cases. Despite these similarities, the neural network-based algorithm runs more than 50 times faster than our previous approach based on HRV features. Although it should be emphasized that the features used in the PPG–HRV classifier were not optimized for computational performance, the type of serial computations used in neural networks is anyway simply much more suitable for small footprint implementations, especially in terms of the number and complexity of mathematical operations performed. The neural network of PPG–NN includes a part for feature extraction (stack of convolutional layers) and a part for inference (recurrent network units). Although in total, the neural network of PPG–NN model uses almost three times more MAC operations than the neural network used for inference with the PPG–HRV algorithm, it is worth noting that in the case of the latter, feature extraction (which is not done by its neural network) takes up 99.5% of the total run time, with the remaining 0.05% being used to infer sleep stages based on a recurrent neural network. Breaking down the computational complexity of PPG–NN, we observe that the feature extraction block is also the more complex, with 79.1 million MAC operations, versus the 10.5 million operations of the recurrent inference block. However, as the difference in run time elucidates, this is still just a small fraction of the time required to compute HRV features, offering a significant advantage from a computational complexity point of view. Furthermore, manufacturers start to support the direct deployment of neural networks in their chips with the expectation that a vast majority of future chipsets will be Artificial Intelligence-enabled^36^. While it is not conceivable that these will support deployment of classical HRV features such as those in our PPG–HRV algorithm, it should be possible to deploy a neural network model such as the PPG–NN on such optimized hardware, which should further reduce its run time and power consumption.

Focusing on the performance of the neural network-based sleep staging algorithm, with a median κ of 0.638, we achieve a higher performance than most work reported in this field^3^, with the added notion that we used a validation set comprising patients with sleep disorders. We observed very few errors between very disparate classes, such as between N3 and REM, or between Wake and N3, or Wake and REM. Most confusion still occurs between N1 and N2 and each of the other sleep stages. This is likely due to the transitional nature of these stages (in particular of N1): inaccuracies in the detection of the exact instant that Wake transitions to N1 or N2, or when N3 starts after a bout of N2, or even in the exact timing and duration of non-REM intrusions in REM periods, all contribute to the increased confusion with this class. Note that the sleep stages N1 and N2 are also the most ambiguous stages when humans score sleep stages using neurological signals according to the AASM scoring rules. Analyses of the AASM interscorer reliability program indicate that 32.4% of all epochs scored as N3 by the majority of scorers were scored as N1 or N2 by individual scorers, 13.8% of all epochs scored as Wake by the majority of scorers were scored as N1 or N2 by individual scorers, and 8.3% of all epochs scored as REM by the majority of scorers were scored as N1 or N2 by individual scorers, while very low numbers of confusions occurred between Wake and N3, Wake and REM and N3 and REM^37^. Thus, the higher number of confusions between the combined N1–N2 class and other sleep stages seems inherent to the task of sleep classification and not a limitation of sleep staging algorithms using autonomic features. Despite this, one cannot exclude the possibility that the increased confusion with N2 might be related to the imbalance between the most frequent N1–N2 class in our training set, which comprises more than 50% of the data, and the less frequent sleep stages in the training set. Although care was taken to choose a loss function that should be somewhat robust to the presence of small imbalances in the distribution between classes, future work could explore whether methods to control class imbalance, like class weighting, or Focal Loss as introduced by Lin et al^38^, could help further increase classification performance. To validate the architectural choices made when developing the neural network in the present study, future work could also explore whether it outperforms other approaches, including transfer learning of networks trained for comparable inputs: the translation from ECG to PPG, for instance, was explored in a previous study, using HRV features as input^39^.

Overall, simple demographic characteristics apart from age do not explain the variance in performance, and neither does AHI, nor the presence of intense body movements and consequent percentage of epochs with valid IBIs. With a pseudo-R^2^ of 0.150 it seems that other, untested factors are driving the differences in performance between participants. Despite the low explanatory power of the variables considered, we did find a significant difference in performance between age groups: the algorithm performs best for adolescents, and worst for adults. We speculate that in the adolescent group (as compared with pre-adolescent children), the sleep architecture, and perhaps more importantly, the HRV expression of autonomic nervous system activity—such as respiratory sinus arrhythmia—is fully established^40^, whereas it is well-known that this phenomenon decreases with increasing age^41^ partly explaining the slight degradation of overall performance.

Regarding the different sleep disorders in our cohort, the algorithm performed significantly worse for patients with movement disorders, with a median κ of 0.591. A more in-depth analysis of this group shows that although age does not seem to explain performance, the presence of large body movements, and—because of the associated artifacts—the percentage of epochs with valid IBIs, both do. With a pseudo-R^2^ of 0.57, these factors seem to play a more important role in explaining the variance in performance in patients with this sleep disorder, which had the lowest overall performance of the entire cohort.

Interestingly, and in contrast with our previous studies that used ECG-^18^ and PPG-based HRV features^3^, the group with REM parasomnias did not perform significantly worse than the group without this condition. We speculate that the set of hand-crafted HRV features used in the previous work failed to capture the possibly different relation between sleep and autonomic nervous system activity in this population e.g., due to a degree of autonomic dysfunction; on the contrary, the neural network, using examples of such patients in the training set, might have succeeded at suitably mapping these relations.

Although the popularity and availability of wearable devices that can—in theory—accurately measure sleep continues to increase, the medical applications of this technology remain elusive, and clinical adoption low as of yet. Nevertheless, the technology continues to mature, and importantly, more evidence becomes available describing its potential, but also limitations. Given its surrogate nature, it will probably never reach a sufficient agreement with gold standard to replace polysomnography; in addition, its use in classical sleep diagnostics will probably remain limited since (for now) it has only been proven to give a reliable measure of sleep architecture but no insights into, for example, the occurrence of arousals or sleep disordered breathing events. But objectively assessing sleep (and wake) even in the presence of insomnia, the possibility of measuring night to night variations in sleep architecture, and of measuring the impact of sleep-related interventions like OSA therapy, may open new avenues of research, leading to a better understanding of how this technology can be used in clinical practice.

## Supplementary Information

Supplementary Information.

## Data Availability

The SOMNIA and HealthBed data used in this study are available from the Sleep Medicine Centre Kempenhaeghe upon reasonable request. The data can be requested by presenting a scientific research question and by fulfilling all the regulations concerning the sharing of the human data. The details of the agreement will depend on the purpose of the data request and the entity that is requesting the data (e.g. research institute or corporate). Each request will be evaluated by the Kempenhaeghe Research Board and, depending on the request, approval from independent medical ethical committee might be required. Specific restrictions apply to the availability of the data collected with sensors not comprised in the standard PSG set-up, since these sensors are used under license and are not publicly available. These data are however available from the authors upon reasonable request and with permission of the licensors. For inquiries regarding availability, please contact Merel van Gilst (M.M.v.Gilst@tue.nl). The SIESTA and the Somnolyzer validation study datasets and the Healthy dataset were acquired or created by Philips Electronics North America and Philips Research Netherlands, respectively, to test automatic sleep scoring software. The three datasets can be used for research purposes within Philips Research. Philips Electronics holds the ownership of the three datasets and these can be shared only under a loan and license agreement. For inquiries, please contact Pedro Fonseca (pedro.fonseca@philips.com).
