# Supplementary material for: A computationally efficient algorithm for wearable sleep staging in clinical populations
Source: Sci Rep. 2023 Jun 6;13:9182. doi: 10.1038/s41598-023-36444-2 (PMC10244431; doi:10.1038/s41598-023-36444-2)
Supplement: Supplementary file 1 — Supplementary Information. [file 41598_2023_36444_MOESM1_ESM.pdf]

Supplementary Materials

to "A computationally efficient algorithm for wearable sleep staging in clinical populations"

Pedro Fonseca[1,2,*], Marco Ross[2,3], Andreas Cerny[3], Peter Anderer[3], Fokke van Meulen[2,4], Hennie Janssen[4], Angelique Pijpers[4], Sylvie Dujardin[4], Pauline van Hirtum[4], Merel van Gilst[2,4], Sebastiaan Overeem[2,4]

[1]Philips Research, Eindhoven, the Netherlands

[2]Department of Electrical Engineering, Eindhoven University of Technology, Eindhoven, the Netherlands

[3]Sleep and Respiratory Care, Philips Austria GmbH, Vienna, Austria

[4]Sleep Medicine Center Kempenhaeghe, Heeze, the Netherlands

*Corresponding author; Pedro Fonseca, Philips Research Eindhoven, High Tech Campus, Eindhoven 34 5656AE, The Netherlands. Email: pedro.fonseca@philips.com.

Table S1 – PSG sleep statistics for all participants in the training and hold-out validation datasets.

Dataset	TST (min)	SOL (min)	WASO (min)	N1 (%)	N2 (%)	N3 (%)	REM (%)	Sleep efficiency (%)
**Training**	**398.5 {347.0, 437.5}<br>[36.5, 606.0]**	**15.5 {7.5, 31.0}<br>[0.0, 426.0]**	**56.0 {30.0, 93.5}<br>[1.5, 404.0]**	**11.2 {7.6, 17.2}<br>[0.9, 77.1]**	**54.0 {48.0, 59.8}<br>[13.7, 92.6]**	**14.7 {8.1, 20.3}<br>[0.0, 49.5]**	**17.9 {14.0, 21.7}<br>[0.0, 45.0]**	**82.4 {72.6, 89.8}<br>[9.4, 99.1]**
SIESTA	397.0 {349.4, 431.5}<br>[70.0, 533.0]	16.5 {8.5, 28.2}<br>[0.0, 209.5]	52.5 {27.4, 91.5}<br>[1.5, 404.0]	10.9 {7.4, 16.6}<br>[2.5, 77.1]	54.0 {48.2, 60.0}<br>[13.7, 79.3]	14.4 {7.9, 19.1}<br>[0.0, 44.5]	18.8 {14.9, 22.1}<br>[0.0, 34.8]	82.8 {74.4, 90.2}<br>[14.6, 99.1]
Somnolyzer validation study	334.0 {284.0, 371.0}<br>[94.0, 462.5]	49.5 {18.5, 89.5}<br>[0.0, 319.0]	63.5 {34.5, 105.5}<br>[4.0, 208.5]	11.1 {6.4, 18.6}<br>[0.9, 57.3]	60.4 (13.4)<br>[26.8, 89.4]	1.7 {0.0, 11.7}<br>[0.0, 33.4]	18.4 (9.3)<br>[0.0, 45.0]	70.8 {63.9, 82.1}<br>[20.7, 95.5]
SOMNIA training	412.5 {361.0, 452.8}<br>[36.5, 606.0]	12.5 {6.5, 26.5}<br>[0.0, 426.0]	61.5 {34.0, 99.8}<br>[3.5, 311.5]	12.4 {8.4, 18.1}<br>[1.0, 60.1]	53.5 {47.8, 58.5}<br>[29.4, 92.6]	16.4 {11.1, 21.1}<br>[0.0, 49.5]	16.8 {13.0, 20.4}<br>[0.0, 31.8]	82.5 {73.8, 89.6}<br>[9.4, 97.9]
Adults	412.2 {358.2, 451.4}<br>[36.5, 580.5]	12.5 {6.4, 26.6}<br>[0.0, 426.0]	63.0 {34.4, 100.5}<br>[3.5, 311.5]	12.5 {8.5, 18.3}<br>[1.0, 60.1]	53.5 {47.9, 58.5}<br>[29.4, 92.6]	16.0 {11.0, 20.8}<br>[0.0, 49.5]	16.7 {13.0, 20.4}<br>[0.0, 31.8]	82.4 {73.0, 89.3}<br>[9.4, 97.9]
Children/adolesc.	487.0 (81.7)<br>[391.0, 606.0]	16.5 (13.1)<br>[3.5, 43.0]	14.5 {10.5, 65.2}<br>[6.5, 90.5]	4.9 (2.3)<br>[2.4, 9.1]	50.4 (9.5)<br>[37.3, 62.0]	28.0 (6.6)<br>[18.4, 37.6]	16.1 (6.1)<br>[5.9, 23.3]	89.1 (9.0)<br>[74.9, 96.7]
HealthBed	444.7 (38.2)<br>[361.0, 510.0]	7.0 {2.0, 10.5}<br>[0.0, 66.5]	34.0 {14.5, 56.5}<br>[5.5, 111.5]	8.3 (3.6)<br>[3.2, 18.4]	48.2 (6.8)<br>[30.4, 60.8]	24.0 (7.3)<br>[9.4, 40.2]	19.4 (4.2)<br>[11.6, 26.8]	91.9 {86.7, 95.9}<br>[74.3, 98.4]
**Hold-out validation**	**419.2 {376.9, 461.0}<br>[102.0, 651.5]***	**14.0 {7.5, 24.5}<br>[0.0, 221.0]**	**44.8 {21.5, 85.5}<br>[2.0, 391.0]***	**11.3 {7.5, 16.3}<br>[1.7, 56.4]**	**49.0 (9.6)<br>[22.2, 78.0]***	**19.7 {14.1, 26.5}<br>[0.0, 66.7]***	**17.8 {13.7, 21.6}<br>[0.0, 38.2]**	**85.5 {76.5, 91.6}<br>[19.0, 99.0]***
SOMNIA hold-out	433.2 {375.8, 469.5}<br>[102.0, 651.5]$^{+}$	13.0 {7.0, 23.5}<br>[0.0, 221.0]	53.2 {24.5, 96.2}<br>[4.5, 391.0]	11.8 {7.5, 17.8}<br>[1.7, 56.4]	51.6 (8.5)<br>[24.8, 78.0]	18.1 {12.3, 23.8}<br>[0.0, 66.7]	16.8 {12.7, 20.4}<br>[0.0, 34.6]	84.7 {74.9, 91.8}<br>[19.0, 99.0]
Adults	419.0 {370.2, 455.6}<br>[102.0, 540.5]	13.0 {6.5, 23.5}<br>[0.0, 221.0]	57.5 {29.0, 101.8}<br>[4.5, 391.0]	13.3 {9.0, 19.2}<br>[1.7, 56.4]	51.7 (8.4)<br>[30.0, 78.0]	17.0 (8.5)<br>[0.0, 40.3]	16.6 {12.7, 20.4}<br>[0.0, 34.6]	84.4 {74.5, 90.8}<br>[19.0, 99.0]
Children/adolesc.	489.5 {438.0, 530.0}<br>[141.0, 651.5]	14.0 {9.4, 24.0}<br>[3.0, 169.5]	26.2 {14.4, 82.6}<br>[6.5, 213.0]	5.3 {3.5, 7.1}<br>[2.4, 17.5]	51.1 (9.1)<br>[24.8, 65.5]	24.4 {19.4, 26.7}<br>[12.3, 66.7]	17.1 (5.7)<br>[0.0, 28.8]	90.4 {78.7, 94.9}<br>[26.9, 97.8]
Healthy	406.0 {380.9, 436.4}<br>[185.0, 520.0]	16.8 {10.1, 28.9}<br>[1.0, 119.5]	29.2 {13.6, 52.4}<br>[2.0, 251.0]	10.4 {7.5, 12.9}<br>[3.0, 30.6]	39.7 {35.0, 49.1}<br>[22.2, 66.6]	26.4 {18.2, 32.8}<br>[10.3, 47.3]	21.3 (5.9)<br>[9.2, 38.2]	87.4 {80.8, 91.0}<br>[36.0, 96.6]

TST: total sleep time, SOL: sleep onset latency, WASO: wake after sleep onset, N1, N2, N3 and REM: percentage of each sleep stage, relative to total sleep time. *, $^{+}$: Significant difference between *hold-out validation and training set, and $^{+}$between SOMNIA subset in the hold-out and SOMNIA subset in the training set, after a Mann-Whitney U test with Bonferroni correction for 20 repeated tests (target p-value for significance = 0.0025). Descriptive statistics indicate mean and between parenthesis, SD in case of normally distributed data, or median and between curly brackets, 25th and 75th percentiles otherwise; the range is indicated between square brackets in both cases. Rows with 'Training' and 'hold-out validation' datasets indicate the aggregated statistics from all recordings on the corresponding subsets. Separate statistics are also provided for each subset and in case of the SOMNIA datasets, further statistics are differentiated between adults and children/adolescents.

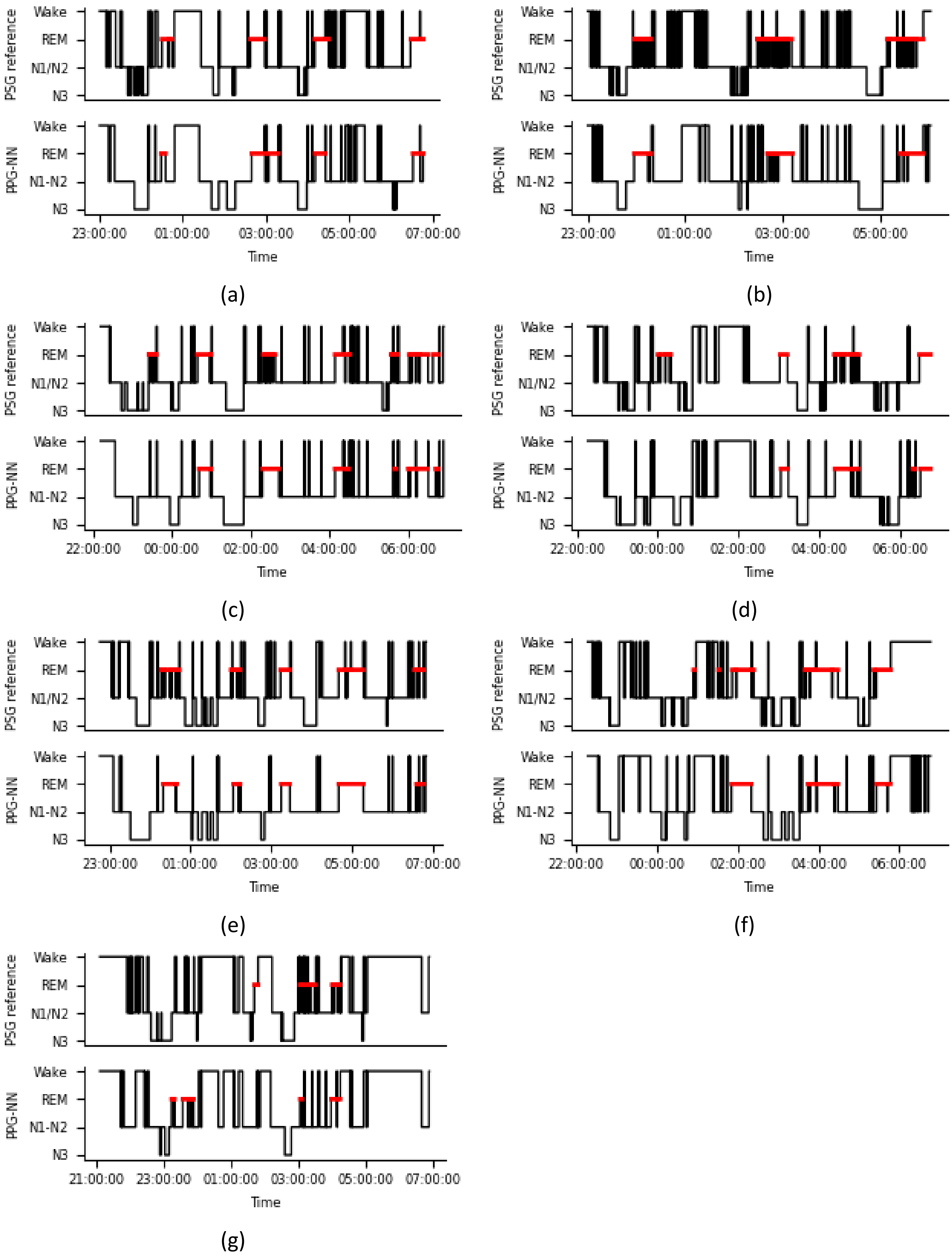

Figure S1 – Example comparisons between reference histogram from PSG and predicted histogram with the PPG-NN algorithm, for the participant closest to the median performance for each sleep disordered category: (a) healthy (κ=0.66), (b) sleep disordered breathing (κ=0.62), (c) insomnia (κ=0.63), (d) movement disorder (κ=0.59), (e) behavioral (κ=0.63), (f) non-REM parasomnia (κ=0.71), and (g) REM parasomnia (κ=0.60).

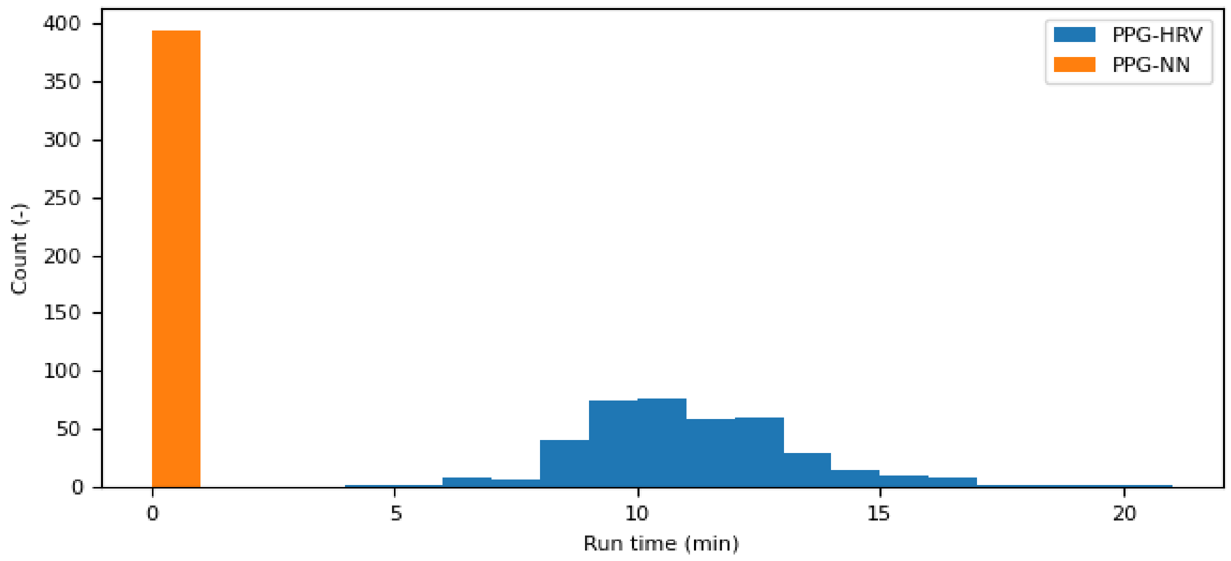

Figure S2 – Histograms with run time per participant (in minutes) for the PPG-HRV and the PPG-NN sleep staging algorithms.

Table S2 – Run time for different feature extraction and inference processing tasks for the recording with the run time closest to the median using with the PPG-HRV algorithm.

**Processing task**	**Time (ms)**	**Time (min)**	**Prct. total time (%)**
Time-domain characteristics including sample statistics of IBIs and instantaneous heart rate and differences between consecutive IBIs[6–8]	4655	0.1	0.7
Frequency domain features, such as the power in the very low, low, and high frequency bands and ratios between these[8,9], with fixed and adapted[10] frequency boundaries	195270	3.3	30.3
Entropy and regularity of IBIs, such as multiscale entropy calculated on windows of 510 s[11]	9039	0.2	1.4
Teager energy of transition points and local maxima in the IBI series[12]	15742	0.3	2.4
Sample entropy of the sign of consecutive IBI differences[13]	299821	5.0	46.6
Visibility graphs and difference visibility graphs, used to calculate the assortativity mixing coefficient, sample statistics of the clustering coefficients and degrees, slope of the power-law fit to the degree distribution, and percentage of nodes with a small and with a high degree[14]	6075	0.1	0.9
Detrended, windowed detrended, and progressive detrended fluctuation analysis of IBI intervals based on sequences of 64 consecutive heart beats[15–17]	91846	1.5	14.3
Cardiac phase synchronization, determined by matching regular patterns in the sign of eight consecutive IBI differences and the occurrence of fixed patterns, dominant rates, and short- and long-term phase coordination patterns[18,19]	9861	0.2	1.5
Higuchi's fractal dimension of normalized IBI series[20]	2533	0.0	0.4
Sample statistics of arousal likelihoods based on sequences of five consecutive heart beats[21]	5628	0.1	0.9
**Total feature extraction**	**640469**	**10.7**	**99.5**
**Inference**	**2951**	**0.0**	**0.5**
**Total**	**643420**	**10.7**	

Table S3 – Mean sleep stage classification performance for several classification tasks in the hold-out validation set (N=394 recordings).

Task	κ (-)	Accuracy (%)	Sensitivity (%)	Specificity (%)	PPV (%)	F1 (%)
**Wake/N1-2/N3/REM**	0.622 (0.124)	76.7 (7.63)	n/a	n/a	n/a	n/a
**Wake/NREM/REM**	0.694 (0.130)	86.2 (6.29)	n/a	n/a	n/a	n/a
**Wake/Sleep**	0.687 (0.152)	92.2 (5.85)	75.8 (15.8)	95.3 (5.57)	75.9 (17.7)	73.7 (13.8)
**N1-2**	0.540 (0.139)	77.6 (6.97)	83.3 (9.20)	72.1 (10.5)	74.7 (12.1)	78.1 (8.92)
**N3**	0.596 (0.209)	90.2 (5.16)	65.5 (23.6)	96.0 (4.16)	74.7 (24.8)	66.0 (18.3)
**REM**	0.696 (0.197)	93.4 (3.76)	71.7 (20.7)	97.2 (2.27)	79.7 (18.1)	74.9 (15.2)

Descriptive statistics indicate mean and between parenthesis, standard deviation. Note that not all variables are normally distributed.

Table S4 – Coefficients and corresponding p-values after beta regression fit to κ with a log-log link function, for all participants, and for sub-groups with movement disorders.

Term	All		Movement disorders	
	**Coefficient**	**p-value**	**Coefficient**	**p-value**
Sex (Male)	-0.0456	0.42	-	-
BMI	0.00278	0.60	-	-
Age	-0.00712	3.9e-05*	-0.00912	0.021
Coverage	0.432	0.035	4.69	4.6e-05*
Act >40	-0.236	0.38	5.10	0.00071*
AHI	-0.00224	0.16	-	-
Pseudo-R^2	0.150		0.565	

Significant terms, considering a Bonferroni corrected p-value of 0.0083 are indicated with an asterisk *.
Although the interpretation of the coefficients, because we use a log-log link function, is not straightforward, the sign of the coefficient indicates whether an increase in that term leads to an increase (if positive) or decrease (if negative) in κ.

Table S5 – Performance on 4-class sleep stage classification on the hold-out validation set, for each age group

Age group	N	κ (-)	p-value	Accuracy (%)	p-value
Children	22	0.650 (0.110)	0.32	0.775 (0.0705)	0.65
Adolescents	26	0.684 {0.627, 0.754}	0.0064*	0.814 {0.781, 0.848}	0.0062*
Adults	346	0.632 {0.566, 0.698}	0.0055*	0.774 {0.731, 0.815}	0.017*

‘Children’ comprises all participants younger than 13, the group ‘Adolescents’ comprises participants older than 13, but younger than 18, and ‘Adults’ all remaining participants. *Significant difference in performance vs the participants of other (combined) age groups.

Supplementary references

1. Radha, M. *et al.* Estimating blood pressure trends and the nocturnal dip from photoplethysmography. *Physiol. Meas.* **40**, 025006 (2019).
2. Eerikäinen, L. M. *et al.* Comparison between electrocardiogram- and photoplethysmogram-derived features for atrial fibrillation detection in free-living conditions. *Physiol. Meas.* **39**, 084001 (2018).
3. Kathirvel, P., Sabarimalai Manikandan, M., Prasanna, S. R. M. & Soman, K. P. An efficient R-peak detection based on new nonlinear transformation and first-order Gaussian differentiator. *Cardiovasc. Eng. Technol.* **2**, 408–25 (2011).
4. Fonseca, P., Aarts, R. M., Foussier, J. & Long, X. A novel low-complexity post-processing algorithm for precise QRS localization. *SpringerPlus* **3**, Article number: 376 (2014) (2014).
5. Fonseca, P. *et al.* Automatic sleep staging using heart rate variability, body movements, and recurrent neural networks in a sleep disordered population. *Sleep* zsaa048 (2020) doi:10.1093/sleep/zsaa048.
6. Redmond, J. & Heneghan, C. Cardiorespiratory-based sleep staging in subjects with obstructive sleep apnea. *IEEE Trans. Biomed. Eng.* **53**, 485–96 (2006).
7. Yılmaz, B., Asyalı, M. H., Arıkan, E., Yetkin, S. & Özgen, F. Sleep stage and obstructive apneaic epoch classification using single-lead ECG. *Biomed. Eng. OnLine* **9**, 39 (2010).
8. Task Force of the European Society of Cardiology and the North American Society of Pacing and Electrophysiology. Heart rate variability: standards of measurement, physiologic interpretation, and clinical use. *Eur. Heart J.* **17**, 354–81 (1996).
9. Bušek, P., Vaňková, J., Opavský, J., Salinger, J. & Nevšímalová, S. Spectral analysis of the heart rate variability in sleep. *Physiol. Res.* **54**, 369–76 (2005).
10. Long, X., Fonseca, P., Haakma, R., Aarts, R. M. & Foussier, J. Spectral boundary adaptation on heart rate variability for sleep and wake classification. *Int. J. Artif. Intell. Tools* **23**, 1460002 (2014).
11. Costa, M., Goldberger, A. & Peng, C.-K. Multiscale entropy analysis of complex physiologic time series. *Phys. Rev. Lett.* **89**, 068102 (2002).
12. Kvedalen, E. Signal processing using the Teager Energy Operator and other nonlinear operators. (University of Oslo, 2003).
13. Cysarz, D., Bettermann, H. & van Leeuwen, P. Entropies of short binary sequences in heart period dynamics. *Am. J. Physiol. - Heart Circ. Physiol.* **278**, 2163–72 (2000).
14. Long, X., Fonseca, P., Aarts, R. M., Haakma, R. & Foussier, J. Modeling cardiorespiratory interaction during human sleep with complex networks. *Appl. Phys. Lett.* **105**, 1–4 (2014).
15. Kantelhardt, J. W., Koscielny-Bunde, E., Rego, H. H. A., Havlin, S. & Bunde, A. Detecting long-range correlations with detrended fluctuation analysis. *Phys. Stat. Mech. Its Appl.* **295**, 441–54 (2001).
16. Penzel, T., Kantelhardt, J. W., Grote, L., Peter, J.-H. H. & Bunde, A. Comparison of detrended fluctuation analysis and spectral analysis for heart rate variability in sleep and sleep apnea. *IEEE Trans. Biomed. Eng.* **50**, 1143–51 (2003).
17. Telser, S. *et al.* Can one detect sleep stage transitions for on-line sleep scoring by monitoring the heart rate variability? *Somnologie* **8**, 33–41 (2004).
18. Bettermann, H., Cysarz, D. & Van Leeuwen, P. Detecting cardiorespiratory coordination by respiratory pattern analysis of heart period dynamics - the musical rhythm approach. *Int. J. Bifurc. Chaos* **10**, 2349–60 (2000).
19. Cysarz, D., Bettermann, H., Lange, S., Geue, D. & van Leeuwen, P. A quantitative comparison of different methods to detect cardiorespiratory coordination during night-time sleep. *Biomed. Eng. Online* **3**, 44 (2004).
20. Higuchi, T. Approach to an irregular time series on the basis of the fractal theory. *Phys. Nonlinear Phenom.* **31**, 277–83 (1988).
21. Basner, M., Griefahn, B., Müller, U., Plath, G. & Samel, A. An ECG-based algorithm for the automatic identification of autonomic activations associated with cortical arousal. *Sleep* **30**, 1349–61 (2007).
